# The Book World of Medicine and Science

**Published:** 1903-05-16

**Authors:** 


### The Book World of Medicine and Science.

The Uniform System of Accounts for Hospitals, Charities, Missions, and Public Institutions. By Sir Henry Burdett, K.C.B. Crown 8vo. (The Scientific Press, Limited, Southampton Street, Strand. 103 pages. Price 4s.)

"The Uniform System of Accounts" is too well known to require recommendation, but Sir Henry Burdett has introduced so much fresh matter into the new edition as, in some ways, to make it quite a new book. The older work is familiar to those connected with hospital work and has met with much approbation. All the old features remain, but the book has been largely rewritten, points that have developed during the last few years being introduced, and new "tips" given as to directions in which economies can be effected without loss of efficiency. In addition to this several fresh chapters have been introduced in explanation of points connected with hospital expenditure, which numerous inquiries addressed to the author showed him required further elucidation for their thorough comprehension by some of those in whose interest continued publication of "The System" is chiefly called for. These are the managers of the smaller institutions, nursing homes, orphanages, etc., as well as those members of hospital and other committees who, though not directly concerned with the preparation of accounts, yet desire to be in a position to assure themselves that the system inculcated is thoroughly carried out. By the larger hospitals and institutions, of course, the system is now thoroughly understood, and by most of them has long since been adopted. To those who know how intensely conservative managers of hospitals, etc., tend to be, this fact is the best testimony to the value of the system that could be afforded, and the new matter in the new edition makes the whole thing so easy of comprehension that there will no longer be any excuse for any charitable institution which does not follow suit. The introduction of this system is on the whole perhaps the most valuable service which Sir Henry Burdett has effected in the cause of philanthropy. Its adoption by all institutions is not only feasible but easy, and several of its advantages are very conspicuous. Apart from the fact that under this system awkward points of expenditure cannot be "burked" by those responsible for them, managers themselves know exactly from day to day where money is going, and members of committee at their meetings can put their finger at once on any item which seems unduly high, and call for an explanation. Last, but not least, members of the public when invited to subscribe to any institution, can, with some approach to accuracy, compare its financial administration with that of other applicants for assistance; while those who are already supporters really have some chance of assuring themselves that the best use is being made of their money. The writer of this notice was himself at one time the author of a system, but he bows to this one, and has only one suggestion to make for a future edition. This is that the "averaging" of certain items, which should vary very little per head and per day, should be advocated as affording to committees a ready clue to careful or careless management during the period under consideration. Upon the whole, the feature in the new edition which the writer of this notice most welcomes is the chapter in which the general system is modified so as to be adapted to the work of missionary societies. Those who are acquainted with missionary work do not need to be told that it is exceedingly difficult at present to acquire any real information as to the precise way in which the vast sums of money collected in England for missionary purposes are expended, or to be reminded of the numerous ways in which questionable expenditure seems liable to occur. Those whose acquaintance with missionary work is confined to subscribing to the funds of various societies may not be acquainted with these points, and all such we recommend to study this system themselves and then urge its adoption upon the authorities of those societies in the welfare of which they are interested.

Introduction to Medical Jurisprudence. By Wm. McCallin, M.D., B.Ch., etc. (London: Baillière, Tindall and Cox. 1902. Pp. 150. 8vo. Price 4s. net.)

The author states that in this book an attempt has been made to give the essentials of medical jurisprudence as definitely, and at the same time as briefly, as possible without producing a cram book. If by a "cram book" the author means a book which gives in a small compass, concisely and accurately, the information which is needed by candidates presenting themselves for examination, then we can honestly say that he has succeeded in *not* producing such a book. For this book is made up of a series of bald, and often disconnected statements which convey to the mind of the reader very incomplete and sometimes very inaccurate information. The chapter on insanity is most inadequate, and the various procedures for placing persons of unsound mind under restraint are very imperfectly described. The logical confusion displayed by some of the definitions is almost ludicrous. "Insanity" is "the state of the mind in which . . . the individual is not responsible for his acts;" an illusion is "when a person sees an object which he believes to be something else;" hallucinations are "as when a person believes he hears voices when there is absolute silence." Perhaps the most remarkably illogical of all Dr. McCallin's statements is his remark that "an insane delusion is of such a character that no rational person could believe him (the patient) capable of holding it if he were sane." Other sections of the work are also misleading. It is not correct to say that strychnine is "seldom used by the homicide on account of its very bitter taste," and such general statements as that "in cerebral hæmorrhage there is hemiplegia," and, "in hysteria the patient is a female, the pulse and temperature are probably normal" do not serve the purpose of the author—the facilitation of differential diagnosis in cases of coma. We cannot commend this book to either "students or practitioners."

Lippincott's Pocket Medical Dictionary. Edited by Ryland W. Greene, M.B. (Philadelphia and London: J. B. Lippincott Co. Price 3s.)

This little volume aims at supplying the long-felt need for a pocket medical lexicon which, though handy in size, should contain the principal terms used in medicine and the allied sciences. The pronunciation and definition of every word are clearly stated, but there is no mention of the derivation. As far as our examination goes the work has been carefully and accurately done, and it should prove extremely useful to nurses and others who must be frequently at a loss to understand many of the new terms which constantly appear in current medical literature.

### BOOKS RECEIVED.

Health Resorts Bureau.

"Carlsbad and its Therapeutical Importance."

A. and C. Black.

"Sour Music." By J. N. Carlyle.

Baillière, Tindall and Cox.

